# Motion state-dependent motor learning based on explicit visual feedback is quickly recalled, but is less stable than adaptation to physical perturbations

**DOI:** 10.1152/jn.00520.2021

**Published:** 2022-08-31

**Authors:** Weiwei Zhou, Elizabeth A. Kruse, Rylee Brower, Ryan North, Wilsaan M. Joiner

**Affiliations:** ^1^Department of Neurobiology, Physiology and Behavior, University of California, Davis, California; ^2^Department of Neurology, University of California, Davis, California

**Keywords:** explicit, motor adaptation, perturbation, savings, visual feedback

## Abstract

Recent studies have shown that adaptation to visual feedback perturbations during arm reaching movements involves implicit and explicit learning components. Evidence also suggests that explicit, intentional learning mechanisms are largely responsible for savings—a faster recalibration compared with initial training. However, the extent explicit learning mechanisms facilitate learning and early savings (i.e., the rapid recall of previous performance) for motion state-dependent learning is generally unknown. To address this question, we compared the early savings/recall achieved by two groups of human subjects. One experienced physical perturbations (a velocity-dependent force-field, vFF) to promote adaptation that is thought to be a largely implicit process. The second was only given visual feedback of the required force-velocity relationship; subjects moved in force channels and we provided visual feedback of the lateral force exerted during the movement, as well as the required force pattern based on the movement velocity. Thus, subjects were shown explicit information on the extent the applied temporal pattern of force matched the required velocity-dependent force profile if the force-field perturbation had been applied. After training, both groups experienced a decay and washout period, which was followed by a reexposure block to assess early savings/recall. Although decay was faster for the explicit visual feedback group, the single-trial recall was similar to the physical perturbation group. Thus, compared with visual feedback perturbations, conscious modification of motor output based on motion state-dependent feedback demonstrates rapid recall, but this adjustment is less stable than adaptation based on experiencing the multisensory errors that accompany physical perturbations.

**NEW & NOTEWORTHY** The extent explicit feedback facilitates motion state-dependent changes to motor output is largely unknown. Here, we examined motor adaptation for subjects that experienced physical perturbations and another that made adjustments based on explicit visual feedback information of the required force-velocity relationship. Our results suggest that adjustment of motor output can be based on explicit motion state-dependent information and demonstrates rapid recall, but this learning is less stable than adaptation based on physical perturbations to movement.

## Graphical abstract

**Figure:**
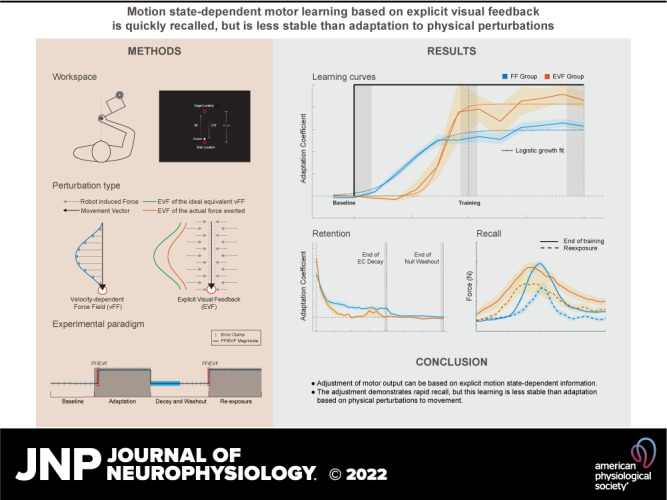

## INTRODUCTION

Motor adaptation is the process of gradually changing movement performance by adjusting behavior in response to changing environmental alterations and perturbations. It is a critical component of how we navigate the world, recalibrating our actions to account for both the self-induced and externally caused movement variability we experience. For example, when walking on ice, one must change gait patterns to maintain balance. Force-field perturbations, an error-based paradigm that applies physical disturbances to movements, are a common method used to systematically study motor adaptation of arm reaching movements (1–7). In the majority of these paradigms, subjects make reaching arm movements using a robotic manipulandum that disrupts their movements with an applied physical force. Adaptation to these force-field perturbations involves motion state-dependent learning, where the temporal force pattern applied by the robot is based on movement state information [e.g., movement velocity or limb position (2, 8–13)]. These disturbances induce motion state-dependent learning—a temporal pattern of motor output (in this case an applied force) based on movement information (e.g., movement velocity or limb position). Thus, over successive trials subjects are able to gradually reduce the force-field induced error by learning the temporal force patterns required to counteract the robot-produced forces (5, 14).

The motor adaptation in the aforementioned examples is based on physical perturbations to movement. In contrast, previous studies of grip force control have shown that subjects can also learn to modulate the temporal patterns of force based on visual feedback (15–20). Object manipulation (e.g., prevention of object slipping or damage) relies on effective grip-load force coupling, where grip force is modulated based upon the experienced load force. In these paradigms, the effect of visual feedback on the accuracy of grip-load force coupling was investigated by having subjects grasp a force transducer coupled to a virtual object and track a moving target. With visual feedback of object motion, subjects showed significant improvement in grip-load force coupling. That is, subjects learned to adjust their grip force in a linear manner with time based on visual feedback of the experienced load force. Although providing direct information has been shown to aid other forms of motor adaptation (21, 22), the extent explicit visual feedback can be used to intentionally learn motion state-dependent force patterns is an open question.

Given the significant role of explicit learning during visuomotor adaptation (23), research has been conducted to evaluate its contribution to adaptation based on dynamic force-field perturbations. Studies have indicated the potential involvement of explicit learning in force-field adaptation using disengagement instructions to abandon learned explicit strategies (24) or to report explicit strategies used during the adjustments to force-field perturbations (25). However, verbal instructions might potentially impact the learning process, and verbal reports have their limitations in characterizing the explicit component. Recently, Schween et al. (26) used a novel approach to access the explicit strategies used during force-field adaptation. The authors showed that subjects can voluntarily control aspects of the force compensation and report their explicit strategies with the untrained limb, providing evidence of explicit learning mechanisms during force-field adaptation. However, as stated earlier, it is unknown if the required changes in the applied force can be acquired based solely on explicit information of the required force-velocity relationship.

Examining the features of motor learning has also provided a way to understand the underlying learning mechanisms, specifically intentional adjustments to motor output. For example, an important property demonstrated for motor adaptation is savings—faster relearning in response to the reexposure to a previously experienced movement perturbation. Savings has been shown for many types of motor perturbations: rotations of movement visual feedback (27–47), saccade target displacements (48, 49), prism displacements (50–52), novel gait patterns (53–56), and novel arm reaching dynamics (57–62). Recent work has demonstrated that early savings of adaptation to force-field perturbations is based on the initial recall of the previous performance (1), where single-trial adaptation (i.e., the temporal patterns of force) following a 24-h break period matched performance at the end of the initial training session. Furthermore, studies using visual perturbations have suggested that savings is largely based on an explicit learning strategy (29, 41), where adaptive changes occur based on conscious, intentional strategies. These studies provide insight into the potential mechanisms that produce early savings/recall of motor adaptation. Yet, the extent adjustments to motor output based on explicit feedback can be recalled is generally unknown.

Here, we examined the extent explicit visual feedback can be used to intentionally learn motion state-dependent force patterns and the subsequent recall of this learning. Rather than parse the contribution of explicit strategies during motor learning to physical perturbations (26), we instead examined the learning and recall achieved by subjects when provided explicit visual feedback information of the required force pattern with no physical perturbation-induced errors. Specifically, we were interested in the extent explicit visual feedback would lead to adjustments in the applied temporal force patterns and the earliest savings achieved (i.e., the recall) after a single trial reexposure to the explicit visual feedback following a decay and washout period. We compared learning, decay, and recall to a control group that experienced physical force-field perturbations. In the experimental condition, participants made error-clamp movements and were given continuous visual representations of both the required force pattern (based on the movement velocity) and their applied lateral force pattern. During training, subjects were instructed to make the two traces overlap. In contrast, the control group experienced physical force-field perturbations and the accompanying multisensory (visual and proprioceptive) errors due to movement disturbance. In both groups, using error-clamp trials, we compared the learning rate, single trial early savings/recall, and stability of the experimental (explicit visual feedback) learning group to the standard (force-field) learning control group. Based on the previous findings from visuomotor rotation (VMR) studies that suggest the majority of savings is due to explicit-based learning strategies (29, 32, 41), we hypothesized that the explicit visual feedback learning group could learn the required force-velocity relationship and would not only demonstrate recall but also show similar levels of recall compared with the force-field learning group.

## MATERIALS AND METHODS

### Participants

Forty right-handed participants (25 females; aged 25 ± 4 yr) without known neurological impairments were recruited from the University of California, Davis, community to participate in the study. Handedness of the subjects was measured by the Edinburgh Handedness Inventory (63). All participants were right-hand dominant and used this hand to complete the experiment. Each participant only performed a single experimental paradigm. The study protocol was approved by the University of California, Davis Institutional Review Board, and all participants gave written informed consent.

### Experimental Apparatus

Experiments were conducted using the Kinarm End-Point Lab (BKIN Technologies), a two-jointed robotic manipulandum that records the velocity, position, and force exerted on the handle of the manipulandum arm. Participants were seated in front of the robotic manipulandum at a height where they could grip the right manipulandum handle at their lower chest level and rest their forehead comfortably on the system’s headrest. They gazed into a horizontal mirror display mounted directly under a downward-facing LCD monitor (1,920 × 1,080 pixel resolution and 60 Hz refresh rate) that projected the visual feedback from the task onto the mirror. Participants’ view of the right forearm and hand position was occluded by the mirror and only a solid white circular cursor (0.3 cm in diameter) was shown to match the handle position. During the experiments, the robotic manipulandum continuously measured hand position, velocity, and forces applied by participants at a sampling rate of 1,000 Hz while simultaneously exerting external forces at the handle to probe their adaptation to the external perturbations.

### Experimental Paradigm

Two experiments were designed to assess motor learning with different types of feedback: standard, physical velocity-dependent force-field (vFF) perturbations and explicit visual feedback of the equivalent vFF perturbations if it was actually applied. Forty participants were divided evenly into the force-field (FF) learning group (*n* = 20) and the explicit visual feedback (EVF) learning group (*n* = 20) and each participant only performed a single experimental paradigm. By comparing the results obtained from the two groups, we aimed to evaluate the influence of explicit visual feedback information on the adjustment of motor output (i.e., the applied force) during reaching arm movements and the retention and early savings/recall of this motor learning.

**Figure 1.**
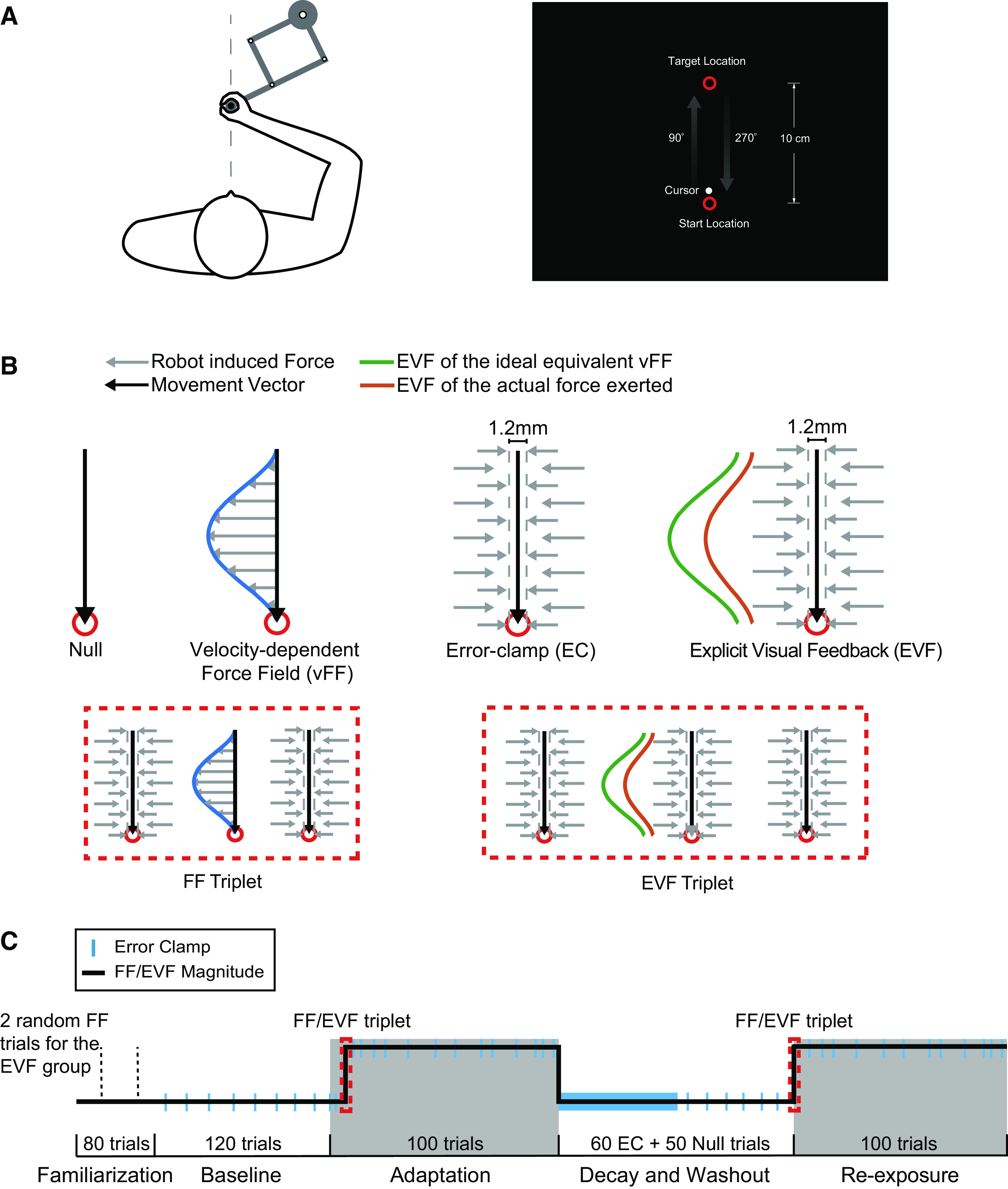
Experimental setup and paradigm. *A*: subjects made reaching movements away (90°) and toward (270°) the body along the midline between two circular targets while holding the handle of the robotic manipulandum. *B*: there were four different trial types used throughout the experiments: null trials, velocity-dependent force field (FF) trials, error-clamp (EC) trials, and explicit visual feedback (EVF) trials. Null trials were movements during which no external force was applied by the robot motors. During FF trials, the robot motors applied a force on the handle that was proportional in magnitude and orthogonal in direction to the velocity of the movement (orange arrow). During EC trials, the robot motors constrained the movements in a straight line toward the target by inhibiting movements perpendicular to the target direction. During the EVF trials, participants were constrained to make straight movements between the two targets with the error-clamp and were shown the visual feedback of the ideal velocity-dependent force-field pattern (dark blue curve) and the actual force pattern participants exerted (light blue curve) in real-time on the screen. The FF triplet and EVF triplet were used to measure the learning in response to the FF perturbation and the EVF, respectively. *C*: the experimental protocol. The subjects in the FF and EVF followed the same basic experimental protocol as the one depicted in the panel except the perturbation trials were different (FF trials for the FF group and EVF trials for the EVF group). In the EVF experiment, subjects briefly experienced two FF trials in the first 80 familiarization trials to measure their hand deviation (dashed lines). This was then compared with two FF trials randomly added at the end of the reexposure period (not shown) to measure how subjects trained with the EVF responded when the FF was actually applied.

For both experiments, participants gripped the handle of the robotic manipulandum and were asked to make rapid point-to-point reaching movements between two red circular targets at the center of the screen positioned at 20 and 30 cm away from the body on the sagittal axis (Fig. 1*A*). To simplify presentation, we refer to movements from the 20 cm target to 30 cm target to be 90° movements, and movements from the 30 cm target to 20 cm target to be 270° movements. The cursor representing the hand position was shown throughout the experiment. In each trial, to encourage participants to make rapid movements, subjects were given visual and auditory feedback about the speed of their movement, indicated by a filled color of green in the end target and a pleasant beep tone when the peak movement velocity was within a range of 0.25–.35 m/s and movement duration was ≤800 ms. Movements above or below the desired range were followed with a fill color of red (too fast) or yellow (too slow), respectively, and no auditory feedback was given.

Four different trial types were used throughout the experiments (see Fig. 1*B*): null trials, FF trials, EVF trials, and error-clamp (EC) trials. Null trials allowed the participant to control the movement of the manipulandum and no external force was applied by the robot’s motors. In EC trials, the robot motors inhibited lateral movements by the subject, maintaining a straight movement trajectory between targets by applying a stiff one-dimensional spring (6 kN/m) and a damper (150 Ns/m) in the axis perpendicular to the reach direction (2, 10, 64, 65). Horizontal hand displacement was limited to <1.2 mm and ∼0.2 mm on average for all participants. By containing participants’ movement in this fashion, the robot was able to keep the lateral errors very small so that the temporal patterns of compensatory force participants applied could be measured independently of feedback responses driven by errors. EC trials were used to assess levels of adaptation throughout the experiments. During FF trials, the robot motors enacted a force on the right manipulandum handle that was proportional in magnitude by a scale of *k* and orthogonal in direction to the velocity of the movement determined by:

(*1*) $$\left[\begin{array}{c} F_{x}\\ F_{y} \end{array}\right]=\;s_{k}\left[\begin{array}{cc} 0 & -k\\ k & \;0 \end{array}\right]\left[\begin{array}{c} \dot{x}\\ \dot{y} \end{array}\right],\;k=15\text{ Ns}/\text{m }$$

where *F_x_* and *F_y_* are the force vectors applied in the horizontal and vertical directions, respectively. $\dot{x}$ and $\dot{y}$ are the corresponding velocity vectors. *s_k_* = +1 and *s_k_* = −1 determined the directions of the force-field: clockwise (CW) or counterclockwise (CCW; an example of the force-field is shown in Fig. 1*B*). For null, FF, and EC trials, only two red circular targets and the cursor were shown on the screen. During the EVF trials, participants were constrained to make straight movement in the force channel between the two targets and the error-clamp was used to measure the lateral force that participants exerted. No physical force-field was applied; instead, we showed the subject the visual feedback of the ideal velocity-dependent force pattern based on the relationship described in *Eq. 1* (dark blue curve shown in Fig. 1*B*) and the actual force pattern participants exerted (light blue curve shown in Fig. 1*B*) in real-time on the screen. The real-time ideal force-field profiles were determined by calculating the force vectors using *Eq. 1* with the instantaneous hand velocity measured by the robot. This was then were plotted as points whose distance to the line connecting the two targets is the force vectors multiplied by a display constant d, where d = 1.5 cm/N (e.g., 1 N force is plotted as a point 1.5 cm away from the midline). The actual force participants exerted was plotted with the same *d* to ensure that the two force profiles shown on the screen were on the same scale. The display constant *d* was chosen to be 1.5 cm/N to provide well-proportioned visual feedback that similarly match the displacements when subjects in the FF group experienced physical perturbations. Due to the high 1,000 Hz sampling rate of the robot, participants could observe continuous curves of the force profiles on the screen. For example, at *t* ms after movement onset, the ideal force-field vector and actual exerted force vector in the range of [0,*t*] ms were calculated and plotted on the screen. On the next frame (*t* + 1 ms), the two vectors in the range of [0, *t* + 1] ms were plotted. The two curves continued to be plotted as subjects made reaching movements. After the subject reached the end target, the two complete force traces stayed on the screen for another 500 ms until the end of the trial. Both FF and EVF were given only on 270° movements, and the directions of the FF and EVF (CW EVF was plotted on the right side of the midline and CCW EVF was plotted on the left side of the midline) was counterbalanced across participants.

### Experimental Task

The structure of the FF experiment was similar to the one proposed in our previous work (1), illustrated in Fig. 1*C*. We instructed participants to make smooth straight movements to the targets and make as many successful movements as possible in both the 90° and 270° reaching directions. The instructions were given before the experiment and were repeated before training and reexposure periods to remind subjects of the goal for their movements. Twenty participants (10 experienced the CW FF and 10 experienced the CCW FF) first completed 80 null trials to become familiar with the experimental setup and desired movement speed. During the baseline period, eight EC trials were dispersed pseudorandomly in 120 null trials to measure the baseline adaptation level before participants experienced the FF perturbations. After the baseline period, participants experienced the adaptation period during which the FF was suddenly introduced after an initial 15 pretraining trials (13 null trials and 2 EC trials). Adaptation trials began with a sequence of EC-FF-EC trials (red dashed box in Fig. 1*C*) to measure the single-trial adaptation to the FF by subtracting the adaptation on the pre-EC trial from the adaptation on the post-EC trial. Participants completed 100 training trials with 20 pseudorandomly interspersed EC trials to measure the adaptation levels during training (after the FF perturbations were introduced, EC trials were applied on all 90° movements). For 270° movements, the ratio of FF-to-EC trials was 2:1 for the first 9 training trials, 5:1 for the middle 84 training trials, and 4:3 for the last 7 trials. The higher ratio used at the beginning and late stages of the training were to ensure an accurate measurement of the initial and final adaptation levels. Immediately following the adaptation period, participants experienced a decay and washout period consisting of 60 consecutive EC trials to measure the adaptation decay and an additional 50 null trials to ensure their performance returned to baseline levels [5 EC trials were pseudorandomly interspersed with these 50 null trials (a 9:1 ratio of null trials to EC trials) to measure the final adaptation levels toward the end of the washout period]. After the washout period, participants were reexposed to the FF perturbations and experienced 100 trials with the same structure as the training trials described earlier. The same EC-FF-EC triplet was used at the beginning of the reexposure period to measure the initial retention and early savings/recall of the initial learning (after a single trial of reexposure).

The EVF experiment was designed following the same basic experimental protocol as the FF experiment (Fig. 1*C*) except the FF trials were replaced with EVF trials. During the EVF trials, participants (*n* = 20, 10 experienced the CW EVF and 10 experienced the CCW EVF) were instructed to make smooth movements and match their actual force profile to the ideal force profile by applying a lateral force on the handle as they made the straight movements between targets, while they saw two curves on the screen as they made reaching movements. If subjects matched the explicit force profiles perfectly, the temporal pattern of the applied lateral force would match the force required to fully compensate for an equivalent velocity-dependent FF perturbation. To ensure the same measurements of learning for the EVF experiment, EC trials were dispersed in the exact same way as in the FF experiment. Consistent with the FF experiment, the instructions were given before the experiment and were repeated before training and reexposure periods. No specific instructions were given to notify subjects about the EC trials. Furthermore, we were interested in the extent participants could compensate for the physical FF perturbations after training with the EVF only. To achieve this goal, we sparsely added two FF trials in the first 80 familiarization trials to measure the hand deviation when participants were briefly exposed to the FF. A high ratio (40:1, null to FF trials) ensured that participants did not learn from these two FF trials as well, which was confirmed by the adaptation levels obtained during the baseline period (see results). In addition, two FF trials were pseudorandomly interspersed in the 15 EVF trials that were added immediately after the reexposure trials at the end of the experiment. We used these two FF trials at the end of the experiment to examine the extent the motor output based solely only the EVF could compensate for the physical FF perturbations. Participants were not informed of the FF perturbations throughout the experiment.

### Data Collection and Statistical Analysis

Throughout both experiments, EC trials (in the 270° direction) were used to measure the lateral forces subjects applied to compensate for the velocity-dependent force-field perturbation (FF group) or match the explicit visual feedback to the ideal force profiles (EVF group). To fully compensate for the vFF perturbation, subjects needed to apply a lateral force that is equal and opposite in magnitude of the ideal perturbation force pattern during movement that was calculated by the movement velocity during the EC trials. For the FF group, numerous studies have shown that the lateral force exerted during the EC trials provides a robust measurement of the predictive feedforward adaptation to the vFF perturbation (3, 4, 11, 66, 67). In the analysis, movement and the corresponding kinematic data for each individual trial were centered on the peak velocity with a temporal window of 1,200 ms (±600 ms, where 0 ms is the moment when the movement reaches peak velocity). This provides an alignment of all movements in the same temporal window. The adaptation coefficient (AC) was used as a metric to quantify the adaptation, which was computed by linearly regressing the lateral force exerted during the EC trials to the ideal force determined by the movement velocity (1–4, 10, 11, 65, 67, 68). To counter any initial biases, mean baseline force profiles (measured from preadaptation ECs placed throughout the baseline blocks) were subtracted from subsequent force profiles on an individual basis (2–4, 10, 11, 65–67). All analyses were performed in the same way for both the FF and EVF groups to ensure consistency and unbiased comparisons.

**Figure 2.**
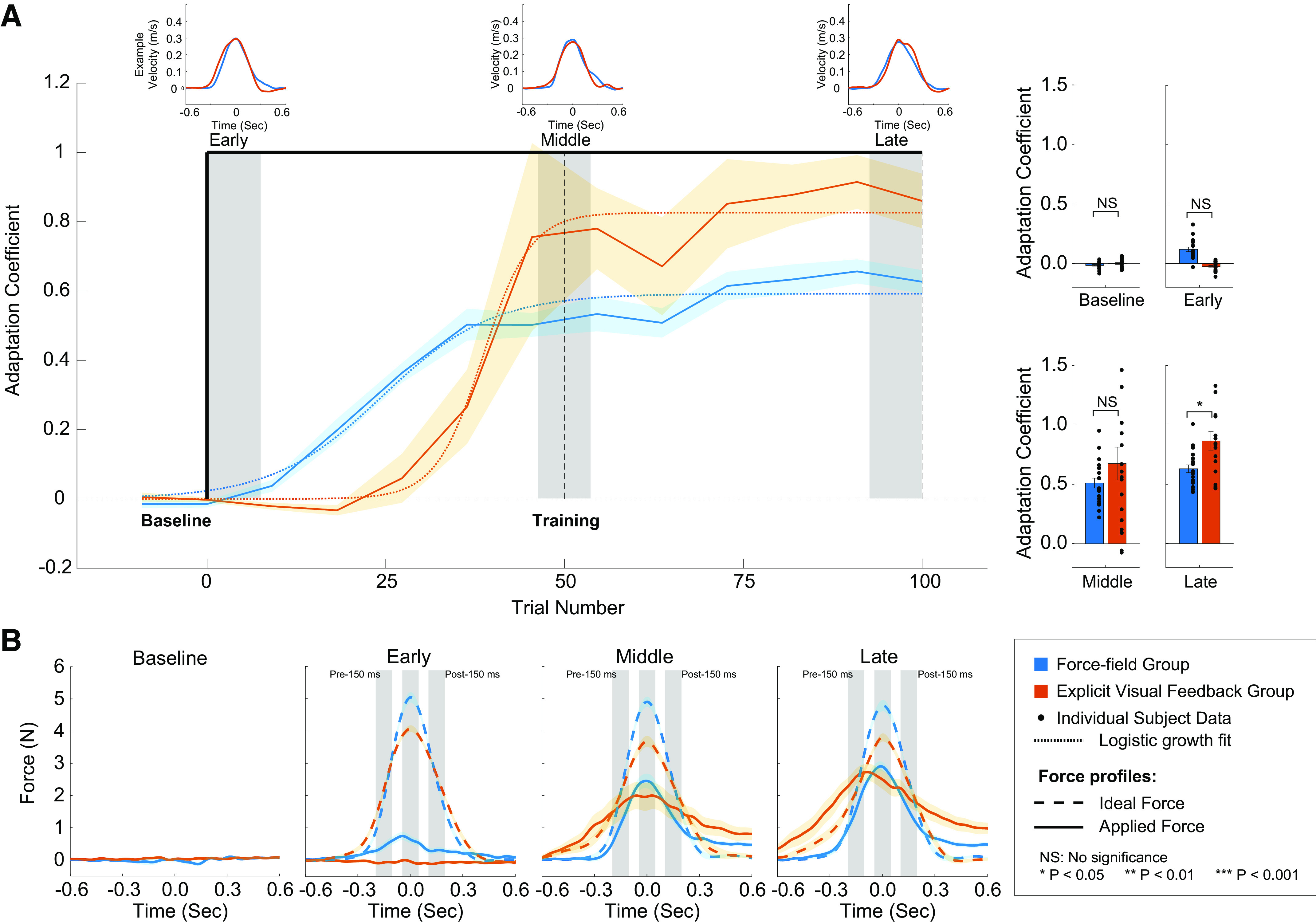
Motor learning during baseline and training blocks. *A*: the adaptation coefficients are plotted as a function of trial number for the force field (FF) and explicit visual feedback (EVF) groups (blue and red traces, respectively). The solid color traces are the mean adaptation coefficient levels, and the shaded color areas represented one SE. The level of the adaptation coefficient for early (the first 10% of training trials), middle (the middle 10% of training trials), and late (the last 10% of training trials) training (shaded gray areas) for the two groups are plotted in the bar graphs to the right. Black dots represent individual data points and the vertical error bars represent one SE. *B*: comparison of temporal force patterns during baseline, early, middle, and late training periods. Solid color traces represent the average applied force of the subjects, and the dashed color traces are the ideal force pattern calculated based on the movement velocity. All force profiles are aligned to the peak velocity. The three gray shaded areas represent the 100-ms window 150 ms before peak velocity, at peak velocity, and 150 m after peak velocity.

As in our previous work (1), the percent recall during reexposure was quantified by determining the slope of the regression (linear mixed-effects model) between the force profile late in training (the last 10 trials of initial training, Fig. 2*B*) and the force profile on the second EC trial of the EC triplet during reexposure (Fig. 5*A*). In this case, the model took the form FP reexposure ∼ FP late training + (1 + FP late training |subject). The random effects of the subjects for the recall were taken into consideration in the model. The regression slope was computed for each subject and scaled by 100 to obtain a percentage of recall.

Data were analyzed offline using Matlab (69) (The MathWorks, Natick, MA) and R 3.6.2 (r-project.org; 70). Trials that had very slow/fast movement speeds (peak speed <0.2 m/s or >0.5 m/s) or abnormal force levels (peak force >15 N or peak force < −5 N) were excluded (∼3% of total trials for FF group and ∼5% of total trials for EVF group). We tested the main effect of group (FF and EVF) on the amount of savings/recall and retention of the adaptation with a linear mixed-effects model (LMM) in R using the *lmerTest* package (71) with the fixed effects of group (FF and EVF) and period (different trial periods in the experiment, e.g., early learning, middle learning, and late learning period; first 10, middle 10, and last 10 training trials, respectively) and random effect of subjects. The model was estimated using the restricted maximum likelihood method (REML) and the significance was obtained using Kenward–Roger and Satterhwaite’s approximations with *pbkrtest* package (72). If significance was identified, post hoc tests were performed using the *emmeans* package and adjusted for multiple comparisons using Bonferroni–Holm corrections. Effect size (*d*) was calculated using Cohen’s *d* (73) measurement (for LMM analysis, generalized effect size was computed following similar procedures of Cohen’s *d* measurement using the *eff_size* function in the *emmeans* package). For all tests, the significance level was set to 0.05. In all cases, group data are presented as means ± SE, and estimation of the proposed model coefficients was reported with 95% confidence intervals. To consider the potential effect of the perturbation directions (CW and CCW) on adaptation, for each LMM model, we included the perturbation direction as a main effect and found no significant main effect or interaction involving the perturbation direction. Thus, the data from CW and CCW perturbations were collapsed together for all of the analysis.

## RESULTS

### Motor Learning: Force-Field Perturbations versus Explicit Visual Feedback

As described earlier, all subjects experienced the same basic experimental paradigm. EC trials were randomly distributed to measure the lateral force profiles subjects applied and adaptation coefficients were determined on these trials to quantify the time course of motor learning (see materials and methods). During the baseline, subject made reaching movements between the two targets without any perturbations/feedback. The average adaptation coefficients (computed over the 8 EC trials during baseline) were not significantly different between subject groups (0.0012 ± 0.0053 for the FF group and 0.0056 ± 0.0051 for the EVF group; two-tailed unpaired sample *t* test, *P* > 0.57, *d* = 0.19) and not significantly different from zero (two-tailed one sample *t* test, *P* > 0.81, *d* = 0.052 for the FF group and *P* > 0.28, *d* = 0.25 for the EVF group).

Before training, two pretraining trials were applied to measure the adaptation coefficient right before exposure to the FF and EVF. Subjects in both groups did not show significantly different coefficient levels from baseline (−0.015 ± 0.0066 for the FF group and 0.0013 ± 0.0070 for the EVF group; two-tailed paired sample *t* test, *P* > 0.11, *d* = 0.59 for the FF group and *P* > 0.68, *d* = 0.16 for the EVF group). The aforementioned results reveal that subjects in the EVF group did not show coefficient levels significantly different from zero, nor significantly different from the FF group in both the baseline and pretraining trials, demonstrating that they did not adapt from the FF perturbations sparsely inserted during the familiarization trials (see materials and methods). To investigate the potential difference in learning, we compared the coefficient after the perturbation was applied/feedback was given (FF or EVF) between the two groups at three different periods during training (early training: first 10 training trials, middle training: middle 10 trials of training, and late training: last 10 training trials). During the entire training period, we observed a fast progression of learning to the given perturbation/feedback information for both groups (Fig. 2*A*). However, during the early, middle, and late training periods, there were differences in the learning levels (adaptation coefficients, early: 0.12 ± 0.019, middle: 0.51 ± 0.041, late: 0.63 ± 0.033 for the FF group; early: −0.027 ± 0.093, middle: 0.67 ± 0.14, late: 0.86 ± 0.077 for the EVF group). An LMM (see materials and methods) was used to investigate the fixed effects of group and training period and random effect of subjects on adaptation coefficients at different periods of training. We found that there was a significant main effect of the training period on learning levels [*F*(2,74.31) = 68.89, *P* < 0.00001] and a significant interaction between the two main effects [*F*(2,74.31) = 5.17, *P* < 0.008]. However, there was no significant main effect of group [*F*(1,39.13) = 1.60, *P* > 0.21]. Post hoc tests were performed and showed that subjects in both groups adjusted to the perturbation/feedback rapidly and their overall learning during the middle training period was significantly greater than the learning during the early training period (*P* = 0.0001, *d* = 1.43 for the FF group and *P* < 0.0001, *d* = 2.55 for the EVF group). All subjects almost reached their asymptotic performance during the middle training period and their learning did not increase significantly between the middle and late training periods (*P* > 0.55, *d* = 0.43 for the FF group and *P* > 0.13, *d* = 0.69 for the EVF group). There was not a significant difference in the adaptation coefficient between the groups during the early and middle training periods (*P* > 0.13, *d* = 0.53 for early training and *P* = 0.10, *d* = 0.59 for middle training). However, subjects in the EVF group demonstrated a higher learning level than the subjects in the FF group during late training (*P* = 0.023, *d* = 0.85).

The temporal force profiles for both groups for the three training periods are shown in Fig. 2*B*. Here, the ideal force pattern (dashed lines) was determined by scaling the temporal vertical velocity profile with the force parameter 15 Ns/m. Interestingly, we found that subjects in the FF group moved significantly faster at peak velocity (early: 0.34 ± 0.01 m/s, middle: 0.33 ± 0.01 m/s, late: 0.32 ± 0.011 m/s) than the subjects in the EVF group during training (early: 0.27 ± 0.0067 m/s, middle: 0.24 ± 0.011 m/s, late: 0.25 ± 0.0081 m/s) which produced a difference between the ideal force patterns of the two subject groups (early: 5.04 ± 0.16 N, middle: 4.89 ± 0.15 m/s, late: 4.77 ± 0.17 for the FF group; early: 4.07 ± 0.10 N, middle: 3.67 ± 0.17 N, late: 3.79 ± 0.12 N for the EVF group). A LMM (fixed effect: group and training period, random effect: subjects) analysis showed that both fixed effects on the peak ideal force were significant [*F*(1,36.88) = 44.79, *P* < 0.00001 for the effect of group, and *F*(2,71.49) = 3.61, *P* < 0.033 for the effect of the training period]. The interaction between the two fixed effects was not significant [*F*(2,71.49) = 0.64, *P* > 0.5]. Post hoc results indicated that for the three training periods, all peak ideal force levels for the FF group were significantly greater than those for the EVF group (*P* < 0.0001, *d* > 1.79 for all cases). This is possibly due to the visual feedback provided to the subjects in the EVF group; requiring subjects to match the two real-time visual force profiles possibly slowed down hand movements. Another possibility could be that subjects slowed down to make matching the two force profiles easier. We tried to minimize these possibilities by repeating the instructions before the training and reexposure periods to remind subjects to move within our desired duration and velocity range (see materials and methods). However, despite the observed differences in the peak ideal force level, the calculation of the adaptation coefficient takes into account these differences in movement speed, allowing a comparison between the two groups.

Our previous study found that measuring the force level 150 ms before and after the mid-movement point provided a good measure of the adaptation specific to a velocity- or position-dependent force-field perturbation, respectively (10). In a similar way, to quantify any potential differences along the temporal force pattern, we analyzed the force data within a 100-ms window centered at peak velocity, and 150 ms before and after the peak velocity point (gray windows in Fig. 2*B*). A LMM was used to investigate the fixed effect of group, training period, and window of force on the temporal force profiles (Table 1). Analysis of the adaptive response within these windows revealed similar results as the analysis on adaptation coefficients; there were no significant effect of subject group [*F*(1,38.14) = 0.0054, *P* > 0.94] nor the interaction among the three fixed effects [*F*(4,289.19) = 1.27, *P* > 0.28]. However, the effects of training period [*F*(2,292.43) = 191.67, *P* < 0.0001], window of force [*F*(2,289.19) = 18.47, *P* < 0.0001], interaction between training period and group [*F*(2,292.43) = 10.00, *P* < 0.000064], interaction between training period and window of force [*F*(4,289.19) = 3.03, *P* < 0.019], and interaction between group and window of force [*F*(2,289.19) = 8.08, *P* < 0.00039] were significant. Post hoc tests showed that there were significant differences in the windowed force at peak velocity during early training (*P* < 0.022, *d* = 0.88) and 150 ms before peak velocity during late training (*P* = 0.0026, *d* = 1.21) between the two groups. Despite these differences, we could see that subjects in the EVF group adjusted the temporal force pattern at a similar rate to subjects in the FF group that experienced the physical vFF perturbations during the three training periods (*P* > 0.063, *d* < 0.66 for all cases). In line with the analysis of adaptation coefficients, subjects in both groups learned quickly from the perturbation/feedback and exerted significantly greater force for all three windows of force during middle training compared with early training (*P* < 0.023, *d* > 0.85 for all cases). The results of the adaptation coefficients and windowed force suggested that subjects in the FF and EVF groups could learn the force-velocity relationship given either real physical perturbations or explicit visual feedback of the required force pattern based on the movement velocity. The EVF group achieved greater learning of the force-velocity relationship from the given explicit visual feedback than the FF group who learned from physical perturbations at the end of the training despite the finding that the peak force levels were similar between the two groups.

**Table 1.** The windowed force (within 100 ms) at 150 ms before the peak velocity, at the peak velocity, and 150 ms after the peak velocity for both the FF and EVF groups during the initial training

	Early Training			Middle Training			Late Training		
	Pre (150 ms Before)	Peak Velocity	Post (150 ms After)	Pre (150 ms Before)	Peak Velocity	Post (150 ms After)	Pre (150 ms Before)	Peak Velocity	Post (150 ms After)
FF	0.45 ± 0.11 N	0.64 ± 0.10 N	0.30 ± 0.078 N	1.12 ± 0.14 N	2.38 ± 0.21 N	1.33 ± 0.15 N	1.62 ± 0.16 N	2.79 ± 0.13 N	1.52 ± 0.10 N
EVF	−0.073 ± 0.036 N	−0.047 ± 0.037 N	−0.094 ± 0.032 N	1.69 ± 0.41 N	1.97 ± 0.41 N	2.55 ± 0.24 N	1.62 ± 0.35 N	2.46 ± 0.27 N	1.94 ± 0.24 N

Data are presented as means ± SE. EVF, explicit visual feedback; FF, force field.

The learning curve for the FF group starts earlier within the first 35 trials and adapted to the physical perturbations in a more gradual way than the EVF group adjusted motor output in response to the visual feedback. The adaptation coefficient for the EVF group increased more abruptly than the FF group, between trials 35 and 50. To better characterize the learning processes for the two groups, we fitted the data with a logistic growth model:

$$A_{n}=\;\frac{K}{1+\left(\frac{K-A_{0}}{A_{0}}\right)e^{-rn}}$$

where *A_n_* is the adaptation coefficient level at trial number *n*, *K* is the carrying capacity (maximum adaptation coefficient level subjects could achieve), *A*_0_ is the initial adaptation coefficient level, and *r* is the growth rate for the learning curve. The fitted learning curves are plotted in Fig. 2*A* (orange dashed line for the EVF group and blue dashed line for the FF group). As seen, the logistic model characterized the learning well for the two groups (*R*^2^ = 0.64 for EVF group and *R*^2^ = 0.72 for the FF group). The carrying capacity for the EVF group is *K*_EVF_ = 0.85 ± 0.089 and learning rate *r*_EVF_ = 1.34 ± 0.79, whereas for the FF group, *K*_FF_ = 0.59 ± 0.029 and *r*_FF_ = 1.18 ± 0.35. These results were consistent with the ones we obtained from the three periods during training. The EVF group learned the required force-velocity relationship given only the visual feedback better than the FF group learned this relationship given the physical perturbations (*K*_EVF_ > *K*_FF_). At the same time, the EVF group reached the asymptote of learning from the initial level slightly faster than the FF group (*r*_EVF_ > *r*_FF_), but the difference was negligible.

### Decay of Adaptation: Force-Field Perturbations versus Explicit Visual Feedback

**Figure 3.**
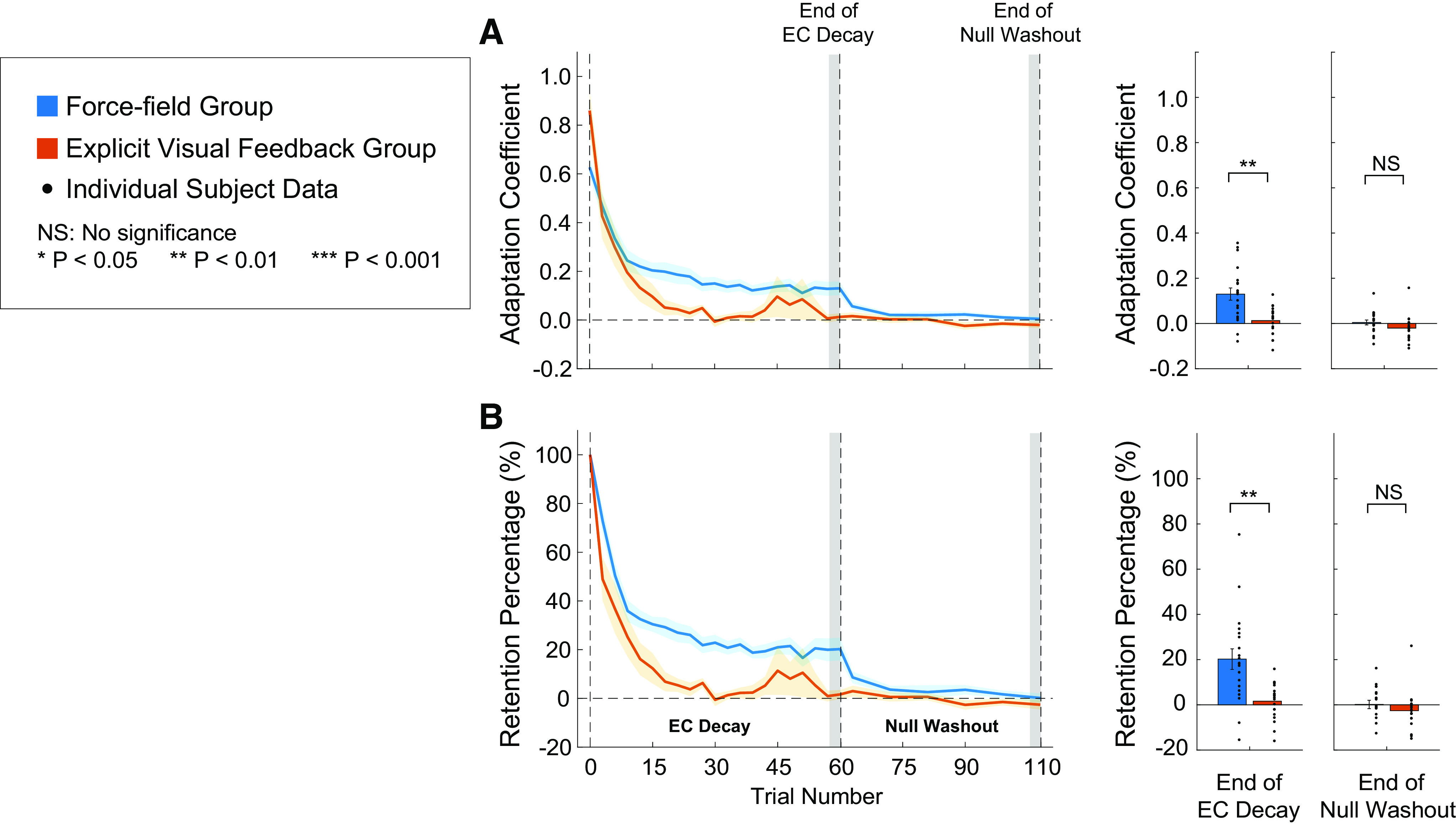
Decay of the adaptation coefficient over consecutive error-clamp (EC) decay trials and null washout trials. *A*: adaptation coefficients are plotted as a function of trials number for the force field (FF) and explicit visual feedback (EVF) groups (blue and red traces, respectively). The solid color traces represent the mean adaptation coefficient levels and the shaded color areas represent one SE. The bar graphs represent the mean adaptation level at the end of the EC decay (last 5% of the EC decay trials, first gray shaded region) and the end of null washout trials (last 5% of the null washout trials, second gray shaded region). *B*: same data shown in *A*, but learning level is represented as a decay percentage (compared with the adaption coefficient level at the end of training) as a function of trial number. The solid color lines are the mean percentage level and the shaded color areas represented one SE. Similar to *A*, the bar graphs show the percentage of learning that remained at the end of the EC decay and null washout trials. In all bar graphs, black dots represent the individual subject data and the vertical error bars represent one SE.

Immediately following the training, subjects in both the FF and EVF groups completed 60 consecutive EC trials to capture the decay of learning. These trials were followed by 50 null trials to washout the remaining learning and return performance to baseline levels. Figure 3*A* shows the decay of the measured adaptation coefficients, and Fig. 3*B* shows the relative decay as a percentage determined by normalizing the adaptation coefficients by the learning level reached at the end of training. We observed a fast decay of the adaptation coefficient for both groups (Fig. 3*A*), with levels decaying over 20% within the first five EC washout trials (Fig. 3*B*). Despite a significantly higher level of learning achieved at the end of training by the EVF group, the adaptation coefficient for the EVF group decayed faster than the learning for the FF group. At the end of the EC decay period (last 5% of the EC decay trials), the retention for the FF group was significantly higher than the EVF group (0.13 ± 0.027 for the FF group and 0.013 ± 0.015 for the EVF group; two-tailed unpaired sample *t* test, *P* = 0.0037, *d* = 1.22). Adaptation coefficient levels decayed further over the null washout trials and returned to levels not significantly different from zero (0.0045 ± 0.01 for the FF group and −0.020 ± 0.013 for the EVF group; two-tailed one sample *t* test, *P* > 0.68, *d* = 0.093 for the FF group and *P* > 0.13, *d* = 0.34 for the EVF group).

For the decay percentage of the adaptation coefficient shown in Fig. 3*B*, we observed similar results; the decay of the learning level over the consecutive ECs for the EVF group was greater (compared with the learning level at the end of training) than the FF group. The percentage of learning at the end of the EC decay period was significantly greater for the FF group compared with the EVF group (20.22 ± 4.54% for the FF group and 1.62 ± 1.87% for the EVF group; two-tailed unpaired sample *t* test, *P* = 0.005, *d* = 1.20). The FF group could still retain ∼20% of their learned adaptation, whereas the EVF group completely abandoned the learned adjustment to motor output after 60 EC trials. As in Fig. 3*A*, at the end of the null washout, the percent learning for subjects in both groups was not significantly different from zero (0.16 ± 1.84% for the FF group and −2.6 ± 2.0% for the EVF group; two-tailed one sample *t* test, *P* > 0.93, *d* = 0.0061 for the FF group and *P* > 0.21, *d* = 0.39 for the EVF group) or between groups (two-tailed unpaired sample *t* test, *P* > 0.32, *d* = 0.33), confirming that at the end of washout period, subjects in both groups returned their performance to baseline before reexposure to the perturbation/feedback. The aforementioned results suggest that learning based on explicit visual feedback of the required motion-state/force relationship was less stable than learning this relationship based on experiencing the physical perturbations.

### Single-Trial Savings/Recall of Motor Learning: Force-Field Perturbations versus Explicit Visual Feedback

**Figure 4.**
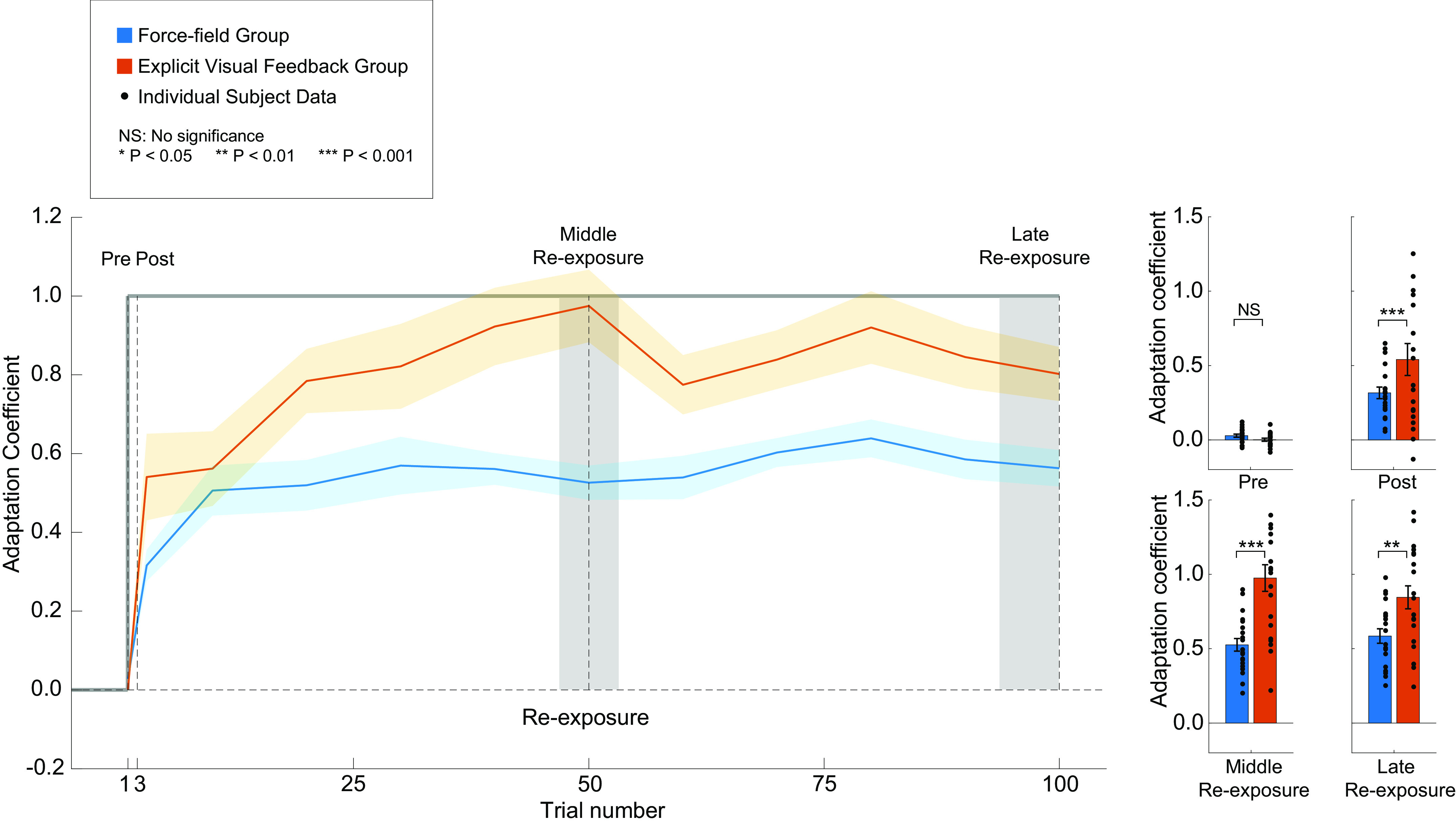
Motor learning during the reexposure period. The adaptation coefficient is plotted as a function of trial number for the force field (FF) and explicit visual feedback (EVF) groups (blue and red traces, respectively). The solid color lines represent the mean adaptation coefficient levels, and the shaded color areas represent one SE. The top bar graphs show the level of learning for the error-clamp (EC) trials immediately preceding and following the single trial of reexposure to the perturbation/visual feedback. The bottom bar graphs show the adaptation coefficient levels for the middle (middle 10% of reexposure trials, first gray shaded region), and late (last 10% of reexposure trials, second gray shaded region) periods during the reexposure for the two subject groups. Black dots represent the individual subject data and the vertical error bars represent one SE.

After the washout period, subjects in the FF and EVF groups were reexposed to the perturbation/visual feedback. The composition of the reexposure block was identical to the initial training block (see Fig. 1*C*). To examine the time course of adaptation savings/learning recall at the beginning of the reexposure, the first three trials consisted of a single trial of the perturbation/visual feedback trial flanked by two EC trials (force-field/visual feedback triplet, see materials and methods). The first EC trial assessed initial retention of the adaptation after washout. After a brief introduction of the perturbation/visual feedback, the second EC trial, when compared with the first, quantified the single-trial adjustment to the perturbation/visual feedback—the earliest assessment of adaptation savings/recall. For the first EC trial, both subject groups had very low adaptation coefficient levels due to the preceding washout (Fig. 4, 0.028 ± 0.011 for the FF group and 0.0014 ± 0.01 for the EVF group). After experiencing only one single perturbation/visual feedback trial, we observed a rapid increase in adaptation coefficient level for both groups on the second EC trial. Interestingly, the EVF group exhibited greater learning than the FF group in this trial (0.32 ± 0.040 for the FF group and 0.54 ± 0.083 for the EVF group). Analysis using an LMM (see materials and methods) with fixed effects of group and EC trials (pre-EC and post-EC trials) and random effects of subjects revealed that there was significant interaction between the two fixed effects [*F*(1,115.24) = 13.79, *P* < 0.001]. Post hoc tests showed that there was no significant difference in the first pre-EC trial between the two groups (*P* > 0.54, *d* = 0.14). However, subjects in both groups rapidly recalled the learned relationship after the brief reintroduction of the perturbation/visual feedback (*P* < 0.0001, *d* = 1.52 for the FF group and *P* < 0.0001, *d* = 2.72 for the EVF group), and the EVF group had a significantly greater adaptation coefficient compared with the FF group (*P* < 0.001, *d* = 1.06), suggesting greater recall. Following the force-field/visual feedback triplet, subjects in both groups rapidly reached their asymptotic performance in the middle of the reexposure (0.53 ± 0.043 for the FF group and 0.97 ± 0.090 for the EVF group). By the end of the reexposure period, both groups had similar learning levels to their learning levels at the end of initial training (0.59 ± 0.049 for the FF group and 0.85 ± 0.077 for the EVF group). Analysis of the adaptation coefficients using an LMM with fixed effects of group and experimental period (the end of the initial training and reexposure period) and random effects of subjects showed that there was significant effect of group [*F*(1,116.46) = 32.14, *P* < 0.0001], but there was no significant effect of experimental period [*F*(1,38.69) = 0.80, *P* > 0.38] nor an interaction between the two effects [*F*(1,116.46) = 1.00, *P* > 0.32].

**Figure 5.**
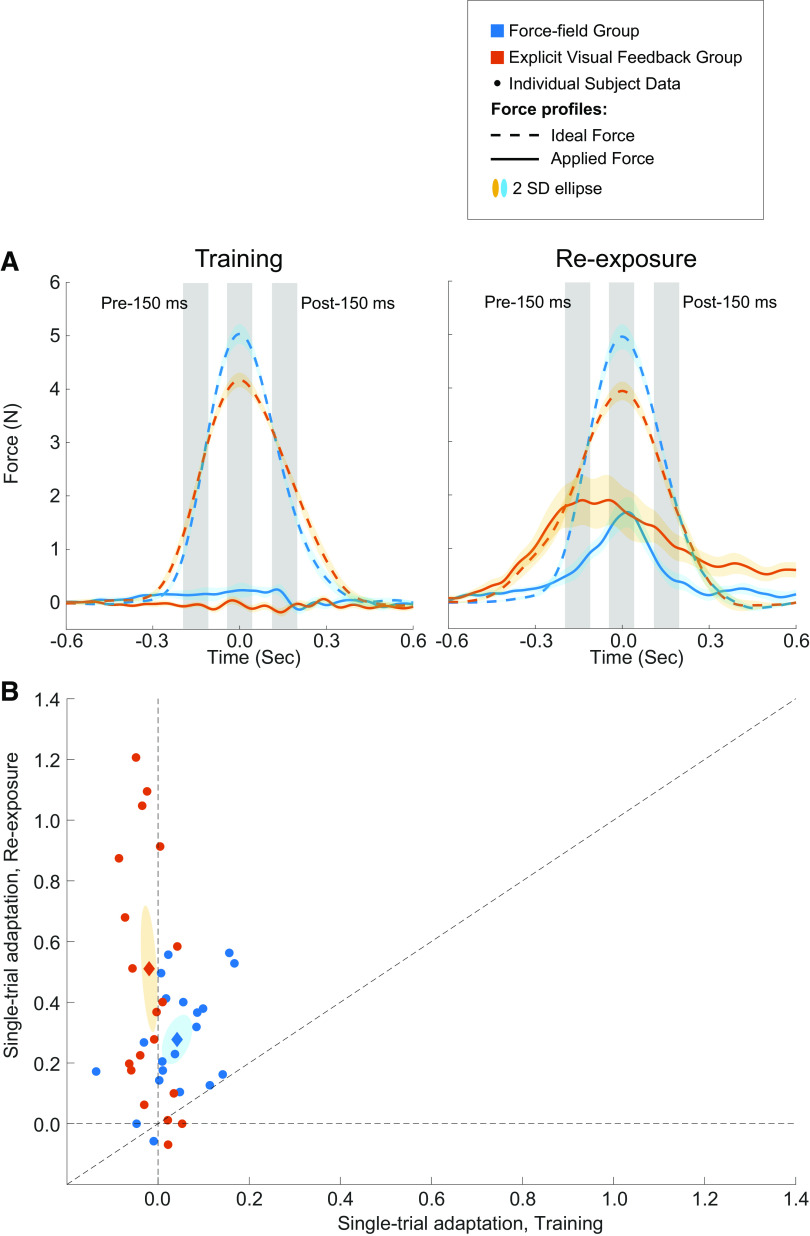
Comparison of single-trial learning during the initial training and reexposure periods. *A*: the force level applied by subjects (solid lines) and ideal force profiles (dashed lines) in the error-clamp (EC) trials immediately preceding and following the single trial exposure to the perturbation/visual feedback during the initial training (*left*) and reexposure periods (*right*). The three gray shaded areas represent the 100-ms window 150 ms before peak velocity, at peak velocity and 150 m after peak velocity. *B*: the single-trial change in the adaptation coefficient during the reexposure period is plotted as function of the single-trial change in the coefficient during the initial training period. Each filled circle represents individual subject data and the filled diamonds represent the group mean values. The shaded ellipses represented two SE.

To better compare the recall achieved by the two groups, the temporal force profiles following the single perturbation/visual feedback exposure in the initial training and reexposure periods are shown for both groups in Fig. 5*A*, respectively. To quantify the differences between the temporal force profiles, we adopted the same approach described earlier and analyzed the force data within a 100-ms window centered at peak velocity, 150 ms before the peak velocity, and 150 ms after the peak velocity point (gray windows in Fig. 5*A*). For both groups, the windowed force after a single FF/EVF trial during the initial training and reexposure is shown in Table 2. Using a LMM with fixed effects of group, window of force and experiment period (the EC trials after a single FF/EVF trial in the initial training and reexposure periods), and random effect of subject, we found significant effects of experiment period [*F*(1,188.23) = 143.91, *P* < 0.0001] and window of force [*F*(2,184.75) = 4.86, *P* = 0.0088] on the force output. The interaction between the effects of group and window of force was also significant [*F*(1,188.23) = 12.24, *P* < 0.0006]. The post hoc tests showed that the windowed force (in all 3 windows) following a single FF/EVF trial in reexposure was significantly greater than the force in the initial training for both groups (*P* < 0.015, *d* > 0.65 for all cases). In the initial training block, the force in the three windows was not significantly different between the two groups (*P* > 0.44, *d* < 0.28 for all cases). However, the EVF group applied a significantly greater force at 150 ms before and after peak velocity following a single EVF trial in reexposure than the FF group (*P* < 0.012, *d* > 0.6), consistent with the analysis of adaptation coefficients.

**Table 2.** The windowed force (within 100 ms) at 150 ms before the peak velocity, at the peak velocity, and 150 ms after the peak velocity for both FF and EVF groups after a single FF/EVF trial during the initial training and reexposure

	Post (150 ms After)		Peak Velocity		Post (150 ms After)	
	Initial Training	Reexposure	Initial Training	Reexposure	Initial Training	Reexposure
FF	0.17 ± 0.08 N	0.83 ± 0.15 N	0.18 ± 0.072 N	1.61 ± 0.24 N	0.023 ± 0.05 N	0.72 ± 0.11 N
EVF	−0.06 ± 0.035 N	1.91 ± 0.43 N	−0.033 ± 0.044 N	1.71 ± 0.43 N	−0.10 ± 0.055 N	1.20 ± 0.26 N

Data are presented as means ± SE. EVF, explicit visual feedback; FF, force field.

To quantify motor learning recall directly, we compared the temporal force profile at the end of initial training (solid red and blue traces in Fig. 2*B*, Late) and the force profile on the second EC trial of the EC triplet during reexposure (solid red and blue traces in Fig. 5*A*, Reexposure) (see materials and methods). (Note that recall is the comparison between the compensatory patterns of force. The adaptation coefficient results presented in Figs. 2, 3, 4, and 5 are a comparison of the compensatory pattern of force and the ideal force pattern based on movement velocity.) As with the adaptation coefficients, the percent recall of motor learning for the explicit visual feedback group was greater than that for the force-field group, but this was not significant (EVF: 54.00 ± 11.28%, FF: 47.73 ± 4.93%, *P* < 0.65).

Finally, we also assessed the initial single-trial learning across groups for both initial training and reexposure blocks that was determined as the difference in the adaptation coefficients between EC trials immediately preceding and following the single trial exposure to the perturbation/visual feedback. Figure 5*B* shows the single-trial learning for reexposure as a function of the single-trial learning for initial training. For both groups, the majority of the individual data (small filled circles) are above the unity line (black dashed diagonal line), demonstrating that the single-trial learning in the reexposure block was greater than the single-trial learning for the initial training block. The majority of the EVF group’s data (filled red circles) and their mean (large filled red diamond) are further away from the unity line than the FF group (blue symbols) signifying the greater recall achieved by the EVF group. Analysis of the single-trial learning using an LMM with fixed effects of group and experimental blocks (training and reexposure) and random effect of subjects showed that there was a significant interaction between the two fixed effects [*F*(1,78) = 5.39, *P* < 0.023]. The post hoc tests showed the single-trial learning was significantly greater in reexposure (0.28 ± 0.041 for the FF group and 0.51 ± 0.10 for the EVF group) than the single-trial learning during initial training (0.072 ± 0.020 for the FF group and 0.026 ± 0.041 for the EVF group) and demonstrated a faster learning rate achieved by both groups (*P* < 0.022, *d* = 0.76 for the FF group and *P* < 0.001, *d* = 1.80 for the EVF group). Even though subjects in both groups had similar single-trial adaptation coefficient levels during initial training (*P* > 0.58, *d* = 0.18), the EVF group had significantly greater single-trial learning in reexposure compared with the FF group (*P* = 0.0078, *d* = 0.86). In summary, the aforementioned results demonstrate learning recall following a single trial of reexposure to the perturbation/visual feedback, but the EVF group showed a greater change in single-trial learning compared with the FF group.

### Adaptation to Explicit Visual Feedback in Response to Physical Perturbations

**Figure 6.**
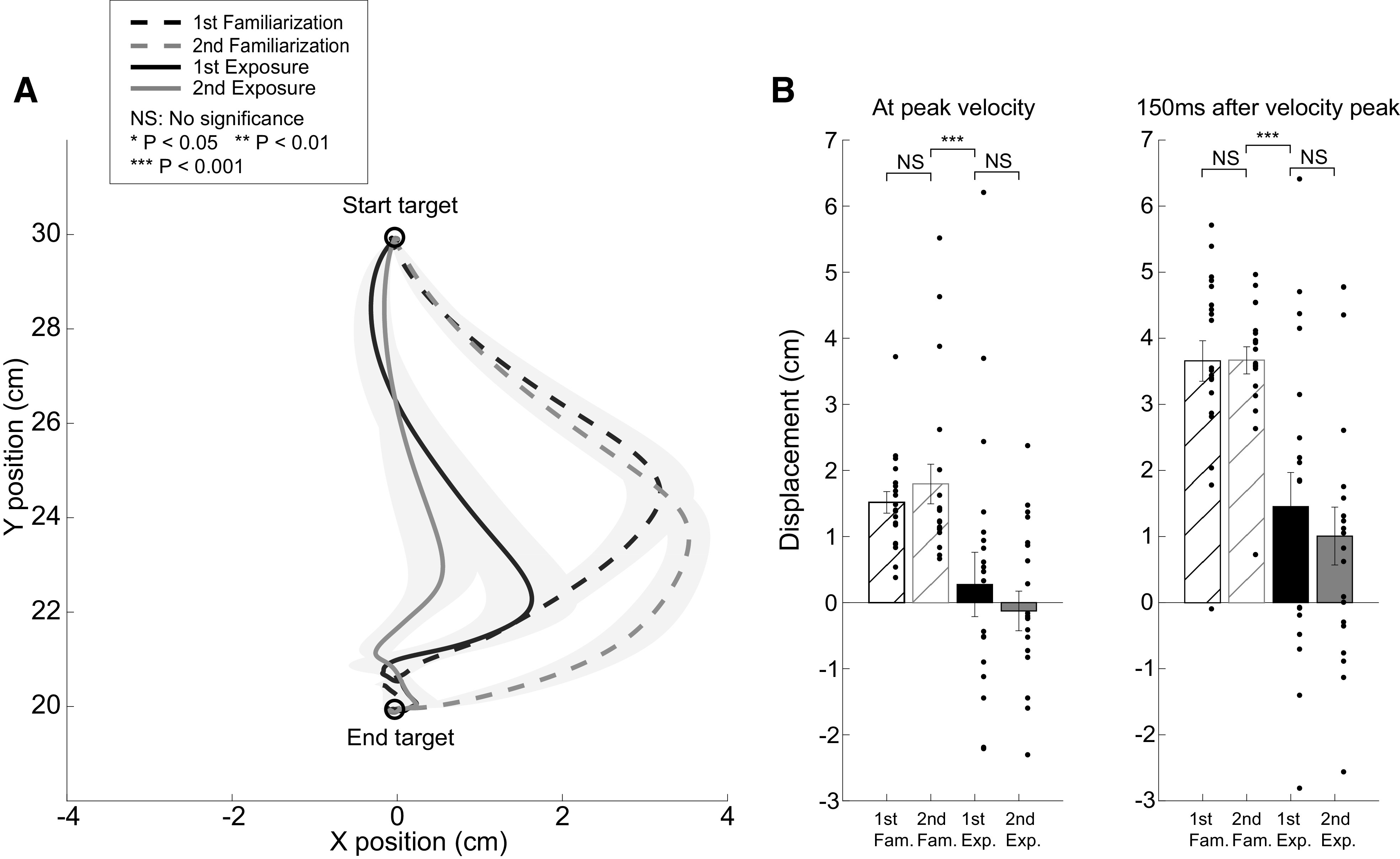
Movement trajectories before and after training with explicit visual feedback (EVF). *A*: average movement trajectories for the subjects in the EVF group when they experienced the force-field during the familiarization (dashed lines) and after reexposure (solid lines) periods. *B*: the perpendicular displacements during familiarization (crossed bars) and after reexposure (filled bars) at the peak velocity and 150 ms after the peak. The displacements after the reexposure period were significantly smaller than those during the familiarization period at the two time points.

The results shown earlier suggest that providing only explicit visual feedback of the required motion-state/force relationship allowed subjects in the EVF group to adjust the applied force during the arm reaching movement to match the pattern required to counter a (not experienced) physical perturbation. These subjects reached a significantly greater learning level than the FF group that experienced the actual force-field perturbations. In addition, the EVF group demonstrated greater single-trial learning of the motor adjustment compared with the FF group after reexposure to the perturbations/visual feedback. This prompts the question: how would the EVF group perform if vFF perturbations were applied following learning based only on the explicit visual feedback? To answer this question, we added a sequence consisting of 15 EVF trials and 2 randomly dispersed FF trials to the end of the reexposure period in EVF experiment. We investigated the performance by comparing the displacements of hand movement trajectories (distance from the line connecting the two targets) on these two FF trials to the displacements on the two FF trials we sparsely applied during the familiarization block to measure subjects’ baseline performance (see materials and methods). As previously described, subjects in the EVF group showed no adaptation at the end of the baseline period after they experienced the two randomly applied FF trials. In Fig. 6*A*, the average trajectories on the two baseline FF trials (black and gray dashed lines) are plotted along with the average trajectories on the two reexposure FF trials (black and gray solid lines). Even when subjects in the EVF group were exposed for the first time to the FF at the end of the reexposure block (solid black trace in Fig. 6*A*), they compensated well for the perturbation; the displacements of the movement trajectories were significantly reduced compared with the two baseline trajectories. On the second exposure of the FF perturbation (solid gray trace in Fig. 6*A*), subjects almost fully compensated for the perturbation. We examined the displacements at two time points: at the peak of the movement velocity (Fig. 6*B*, *left*) and 150 ms after the velocity peak (Fig. 6*B*, *right*). The perturbation resulted in greater displacements later into the movement (at peak velocity: first FF trial during familiarization: 1.52 ± 0.16 cm, second FF trial during familiarization: 1.79 ± 0.30 cm, first FF trial during reexposure: 0.27 ± 0.49 cm, second FF trial during reexposure: −0.13 ± 0.30 cm; 150 ms after peak velocity: first FF trial during familiarization: 3.66 ± 0.31 cm, second FF trial during familiarization: 3.67 ± 0.20 cm, first FF trial during reexposure: 1.45 ± 0.52 cm, second FF trial during reexposure: 1.01 ± 0.44 cm). An LMM was performed to examine the fixed effects of trial type (first and second FF trial during familiarization, first and second FF trial during reexposure) and time-point of displacement (at the peak velocity and 150 ms after the peak velocity point) and random effect of subject on the displacements of hand trajectories. Both fixed effects were significant [*F*(3,133) = 30.26, *P* < 0.0001 for the effect of trial type and *F*(1,133) = 54.97, *P* < 0.0001 for the effect of time-point of displacement] while their interaction was not [*F*(3,133) = 1.39, *P* > 0.24]. Post hoc tests showed that for all four FF trials, displacements at 150 ms after peak velocity point were greater than the displacements at the peak velocity (*P* < 0.0089, *d* > 0.84 for all cases). For both time points of displacement, there was no significant difference in displacements between the first and second FF trial during familiarization (*P* > 0.92, *d* < 0.21 for all cases). The displacements for the first FF trial during reexposure was significantly reduced compared with both FF trial during familiarization (*P* < 0.025, *d* > 0.92 for all cases). On the second FF trial during reexposure, the displacements were further reduced but not significantly different from the first FF trial during reexposure (*P* > 0.9, *d* = 0.30 for velocity peak and *P* > 0.9, *d* = 0.33 for 150 ms after velocity peak). Therefore, when provided with only explicit visual feedback of the required force-velocity relationship, subjects in the EVF group were able to compensate for the real vFF perturbations. Together, the results from the FF and EVF experiments again suggest that subjects can adjust motor output based on explicit visual feedback of the required motion-state/force relationship and demonstrate recall of this learning even without experiencing the movement errors from the physical perturbation.

## DISCUSSION

In this study, we probed motor learning in two main groups of subjects based on the type of information provided. Subjects in the force-field (FF) group experienced standard velocity-dependent force-field perturbations to the reaching arm movement. Subjects in the explicit visual feedback (EVF) group did not experience this physical perturbation of the movement. Instead, on each trial, they moved within a force channel that restricted movement along a set path. During the movement, they were provided visual feedback of the lateral force produced during the movement as well as the ideal force pattern based on the movement velocity. The latter is the temporal pattern of force that the subject would be required to apply to counter a velocity-dependent FF perturbation if it was actually experienced during the movement/trial. Subjects were instructed to match the force profiles in real time during the movement. Our aim was to probe the extent subjects in the EVF group could *1*) learn the correct force pattern to counter the (nonexperienced) FF perturbations given only the visual feedback of the required motion-state/force relationship, *2*) maintain this learning when the feedback was withheld, and *3*) demonstrate recall of learning when the explicit visual feedback was introduced again. Even though the information provided to the two groups was different, the assessment was not; the adaptation coefficient, based on the ideal and applied temporal force profiles, was quantified on force channel trials that were exactly the same for both groups. Thus, we compared performance for the explicit feedback group to the group that experienced the physical movement perturbations to quantify differences in the time course of motor learning and decay, as well as the amount of recall for the learned motion state-dependent changes to motor output.

### Adaptation of Movement Dynamics Based on Visual Information

Numerous studies have used visual information to examine motor adaptation and the savings of the learned motor recalibration. For example, visuomotor rotation (VMR) is a motor adaptation paradigm based on visual feedback perturbations where subjects have to adjust for the discrepancy between the actual arm movement and the visual feedback provided (35, 74, 75). Previous studies using visuomotor rotation paradigms have demonstrated learning and savings is partially based on utilizing explicit knowledge of the motor recalibration (29, 32, 41). Here, we modified the standard force-field adaptation experiment (5) to more closely resemble the visuomotor rotation perturbation. Subjects were required to learn the motion-dependent force relationship, but without any proprioceptive errors—only based on the provided visual feedback of the required temporal force pattern. Thus, we provided direct information of the required change to motor output (21, 22) and were interested in the extent this explicit visual feedback could be used to intentionally learn the required motion state-dependent force patterns. Our experimental group demonstrated recall of the learning upon reexposure after having trained with explicit visual feedback alone. Not only was recall observed in this condition, but it was also similar to that observed in the control group (standard adaptation to physical force-field perturbations). However, we should note that our study was limited in the extent it can quantify the concurrent contribution of both implicit and explicit processes in the adjustment to novel movement dynamics. That is, our study demonstrates that motor learning and recall can be achieved when provided explicit information of the required force pattern with no physical perturbation-induced errors. It does not however isolate the explicit learning contributions during the adaptation to physical FF perturbations for the FF group (26), nor does it separate any implicit learning mechanisms during motor learning based on the explicit visual feedback.

Though much of the literature on the use of explicit visual feedback information to recalibrate motor output is from VMR studies, there have been previous studies in which this feedback is used to modulate grip force patterns (15–20). In these studies, visual feedback was used to measure the impact on learning and the control of grip force. Gibo et al. (15) found that without force feedback subjects were unable to couple grip and load force and that the absence of training with explicit feedback led to a decrease in subsequent performance. Using a similar handheld force transducer paradigm, patients with impairments in hand function following brain lesions of various causes (e.g., traumatic brain injury, viral encephalitic, etc.) successfully reduced their grip force control error after training with visual feedback of the required force (17). These results demonstrate that the visual representation of the required force pattern significantly improves adaptation and learning, similar to previous studies (22) and the results described earlier. Although these force pattern adaptation studies of grip force resemble our experimental design, they did not base the temporal force pattern on motion state (i.e., movement velocity) nor assay learning recall. To our knowledge, there are no other force-field adaptation studies based solely on explicit visual feedback of the required motion-state/force relationship that have measured recall. Matthew et al. (76) recently demonstrated that savings in the context of force-field learning may be due to feedback adaptation rather than an explicit reaiming strategy, although not in the context of explicit feedback of the required force. Thus, although our results suggest recall can result from providing only explicit information of the required force-velocity relationship, there remains an open question on the extent explicit-based learning strategies are the main component of savings for adaptation to novel physical dynamics.

### Contribution of Different Learning Mechanism

The different types of information provided to learn the required force-velocity relationship resulted in different time courses for motor learning (Fig. 2), strengthening the argument that different mechanisms were engaged for both groups. Although the ultimate goal is the same, improvement by the FF group is more of a gradual process than for the EVF group. In fact, the initial rise of the learning curve for the EVF group is delayed compared with the FF group, suggesting that subjects required a number of observations before the correct relationship could be fully applied. In contrast, the FF group demonstrates a steadier, iterative increase in learning starting from the introduction of the perturbation. Despite this difference in how the EVF subjects learned the required force-velocity relationship, the random FF trials applied at the end of training (Fig. 6) provided clear evidence that training with the feedback improved performance in the FF even without experiencing the physical movement errors. Note that the application of these FF trials at the end of the experiment was done to avoid the complications of increased limb stiffness in response to experiencing large motor errors (77–79). In fact, we chose to assess learning and subsequent recall through error clamp trials rather than FF trials to avoid the issue of stiffness. This effect on performance in the FF raises the possibility to examine the synergetic effects, and possible interference, when both types of information are provided during training.

Although the explicit visual feedback group demonstrated similar recall, we observed faster decay of learning for the EVF group compared with the FF group (Fig. 3). These results suggest that learning based on explicit visual feedback of the required motion-state/force relationship was less stable than learning this relationship based on experiencing the physical perturbations. Decay of motor adaptation has been demonstrated with the removal of movement feedback and the passage of time (23, 64, 80–85), consistent with our observation of overall decay in both conditions. However, this does not account for why there is a faster decay among the explicit visual feedback group. As with the learning curves, this difference in decay suggests that different learning mechanisms are being utilized in each condition. Aspects of this may be explained by the two-state sensorimotor learning system (86) that posits there are two distinct learning mechanisms, a fast process and a slow process, and the division of sensorimotor learning into explicit and implicit learning (23). Recent evidence has been found to suggest that these two frameworks may have some overlap: explicit learning may contribute to the fast-learning process and implicit learning may play a role in the slow learning process (58, 87, 88). Early learning has been clearly associated with fast time-scale processes and rebound effects associated with slow processes have been shown later in learning, although the stability of each was not assessed (58). In the present study, the explicit visual feedback group could be adjusting the applied temporal pattern of force based largely on fast, explicit learning processes, whereas the force-field group adapts based on a combination of fast (explicit) and slow (implicit) learning mechanisms. However, this may only be partially true, as is it still unknown (within the context of adaptation to physical perturbations) whether slow and fast learning mechanisms directly overlap onto implicit and explicit learning, respectively.

Another potential (and related) explanation for the difference in learning stability between the EVF and FF groups is that of sensory prediction error and task outcome error. Sensory prediction errors (SPE) are the difference between the expected and observed outcomes following a motor command. In contrast, task outcome errors (TE) are those based solely on information regarding task accuracy, commonly provided through online task performance feedback. Previous studies have suggested that TE drives explicit learning, whereas SPE largely engages implicit learning mechanisms (89, 90). However, recent work has shown that TE may serve as a gain or contribute to implicit learning when provided in parallel with SPE, rather than the two error types inducing distinct learning mechanisms (91, 92). Nevertheless, TE alone was not shown to be sufficient in producing implicit learning (92), although it does engage explicit learning mechanisms. In our design, participants in the EVF group were only provided TE feedback, supporting this theory that it facilitates explicit learning adaptation. The FF group likely utilized a combination of SPE and TE, providing feedback on their accuracy in making a straight movement to the target location. It is likely that the EVF group quickly abandoned the learned explicit strategy when the TE feedback of the perturbation was no longer presented. In contrast, the recalibration of movement in response to the SPE for the FF group was a relatively more stable change; a component of updating of the internal model of limb movement dynamics persisted even when the information was no longer present (93).

### Motor Learning Recall versus Savings

In this study, we were focused on the earliest component of savings, that following a single trial of reexposure. Our previous study (1) showed that a significant amount of adaptation savings for the recalibration of movement in response to force-field perturbations is initially due to the rapid recall of previous performance. We trained subjects for different durations on *day 1* and then examined recall (using the same EC triplet utilized in the current study) on *day 2*. The force profiles on *day 2* revealed a rapid recall of the previously learned motor recalibration; the temporal pattern of force following the first perturbation on *day 2* closely matched that at the end of *day 1*. This recall was highly dependent on the initial training duration on *day 1* and final adaptive state (i.e., if subjects experienced a washout period at the end of *day 1*). In the current study, the recall for the FF and EVF groups accounted for a substantial amount of the motor learning demonstrated during reexposure. That is, following only a single trial of reexposure, subjects exhibited >50% of the total learning level reached during reexposure to the given perturbation/feedback information. Although there was no significant difference in the recall between the two groups, the EVF group demonstrated significantly greater single trial learning compared with the FF group (Fig. 5). The key difference between these two measures (recall vs. single trial learning) is that the former is a comparison of the applied force to the previous force profile at the end of initial training whereas the latter is a comparison of the adaptation coefficient at the start of initial training and reexposure. In addition, the adaptation coefficient is dependent on the applied force and the ideal force profile based on the movement velocity. Due to these differences, it could be argued that the recall is the more accurate comparison between the two subject groups. That is, the relatively lower max velocity and adaptation coefficient levels for the EVF group at the start of initial training amplify the single-trial learning comparison. In addition, the recall metric is a more direct measure of the extent subjects apply the learning reached at the end of initial training. Finally, recall likely probes the influence of explicit learning processes during early savings; the brief single trial exposure makes it unlikely that the motor response is due to an error-driven, implicit learning mechanism. Thus, the motor output following a single trial of reexposure may prompt the rapid execution of previously successful movements (31, 42). Future studies could probe this possibility to determine the extent the single-trial recall can be induced and modulated with nonspecific physical disturbances or feedback information. For example, recall could be assessed for a force disturbance or visual cue different from that experienced during initial training [e.g., a force pulse perturbation (12, 94), or explicit visual feedback in the opposite direction than that experienced during initial training].

### Summary

We have shown that motor learning of force-velocity relationships based on explicit visual feedback alone demonstrates similar recall to motor adaptation based solely on force-field perturbations. However, learning based on this feedback was less stable and decayed at a significantly faster rate. The results are consistent with the idea that explicit, intentional learning can be rapidly recalled, but that this learning decays quickly in the absence of consistent feedback. Future studies may intermix explicit visual feedback with force-field perturbations to probe the influence on learning when provided with the combined information.

## GRANTS

This work was supported by a grant from the National Science Foundation Grant 1553895 (to W. M. Joiner).

## DISCLOSURES

No conflicts of interest, financial or otherwise, are declared by the authors.

## AUTHOR CONTRIBUTIONS

W.Z. and W.M.J. conceived and designed research; R.B., R.N., and W.Z. performed experiments; R.N. and W.Z. analyzed data; W.Z., E.A.K., R.B., and W.M.J. interpreted results of experiments; W.Z., E.A.K., R.B., and W.M.J. drafted manuscript; W.Z., E.A.K., R.B., and W.M.J. edited and revised manuscript; W.Z., E.A.K., and W.M.J. approved final version of manuscript.

## ENDNOTE

At the request of the authors, readers are herein alerted to the fact that additional materials related to this manuscript may be found at https://doi.org/10.6084/m9.figshare.17096630. These materials are not a part of this manuscript and have not undergone peer review by the American Physiological Society (APS). APS and the journal editors take no responsibility for these materials, for the website address, or for any links to or from it.
